# Auditory Stimulation Modulates Resting-State Functional Connectivity in Unresponsive Wakefulness Syndrome Patients

**DOI:** 10.3389/fnins.2021.554194

**Published:** 2021-02-16

**Authors:** Melanie Boltzmann, Simone B. Schmidt, Christoph Gutenbrunner, Joachim K. Krauss, Martin Stangel, Günter U. Höglinger, Claus-W. Wallesch, Thomas F. Münte, Jens D. Rollnik

**Affiliations:** ^1^BDH-Klinik Hessisch Oldendorf, Hessisch Oldendorf, Germany; ^2^Clinic for Rehabilitation Medicine, Hannover Medical School, Hannover, Germany; ^3^Department of Neurosurgery, Hannover Medical School, Hannover, Germany; ^4^Department of Neurology, Hannover Medical School, Hannover, Germany; ^5^BDH-Klinik Elzach, Elzach, Germany; ^6^Department of Neurology, University of Lübeck, Lübeck, Germany

**Keywords:** neurological early rehabilitation, disorders of consciousness, unresponsive wakefulness syndrome, auditory stimulation, resting-state fMRI

## Abstract

Passive listening to music is associated with several psychological and physical benefits in both, healthy and diseased populations. In this fMRI study, we examined whether preferred music has effects on the functional connectivity within resting-state networks related to consciousness. Thirteen patients in unresponsive wakefulness syndrome (UWS) and 18 healthy controls (HC) were enrolled. Both groups were exposed to different auditory stimulation (scanner noise, preferred music, and aversive auditory stimulation). Functional connectivity was analyzed using a seed-based approach. In HC, no differences were found between the three conditions, indicating that their networks are already working at high level. UWS patients showed impaired functional connectivity within all resting-state networks. In addition, functional connectivity of the auditory network was modulated by preferred music and aversive auditory stimulation. Hence, both conditions have the potential to modulate brain activity of UWS patients.

## Introduction

Disorders of consciousness (DoC) refer to an impaired level of awareness of the self and the environment, presenting as coma, unresponsive wakefulness syndrome (UWS), or minimally conscious state (MCS) (Gosseries et al., 2014). While coma is an acute state of unconsciousness and unresponsiveness, that most patients overcome within a few hours or days, some patients develop prolonged disorders of consciousness (Hannawi et al., 2013), such as UWS or MCS. UWS is characterized by preserved eye-opening and sleep-wake-cycles without behavioral signs of self-related or environmental awareness. Some patients further progress to MCS, characterized by inconsistent, but reproducible signs of awareness (Giacino et al., 2002).

Since the auditory modality is particularly sensitive in detecting signs of awareness (Gill-Thwaites and Munday, 1999; Owen et al., 2005; Zhu et al., 2019), auditory stimulation has received great attention over the last years. For example, UWS patients show responses to pain cries of others (Yu et al., 2013), their own name (Perrin et al., 2006; Di et al., 2007; Cheng et al., 2013; Wang et al., 2015), verbal instructions (Owen et al., 2005), and sentences (Coleman et al., 2007). Music is a special type of auditory stimulation that may be beneficial in DoC patients (Kotchoubey et al., 2015). The positive effects of music are attributed to the recovery of specific brain networks required for the processing of sensory input, and to the emotional aspects of music, which might increase arousal and activate the reward system (Perrin et al., 2015). Previous studies have shown that musical activities, including passive listening to music, are associated with several psychological and physical benefits in both healthy and clinical populations (Grimm and Kreutz, 2018). For example, listening to preferred music has the potential to reduce pain and anxiety (Hole et al., 2015) and sedation frequency (Chlan et al., 2013). After stroke, it improves cognitive recovery and mood (Särkämö et al., 2008) and induces structural changes in frontal, medial and limbic areas (Särkämö et al., 2014). Studies with DoC patients also provide promising results [see Grimm and Kreutz (2018) for a review]. As emotionally salient stimulus, preferred music has beneficial effects on cognitive processes and behavioral responses in DoC patients (Raglio et al., 2014; Verger et al., 2014; Choudhry et al., 2016). In a patient with hypoxic-ischemic encephalopathy who was considered to be in UWS, a music therapy assessment revealed purposeful responses, changing the diagnosis to MCS (Magee, 2005). This case illustrates the potential role of music therapy as clinical tool assisting with the diagnosis of patients with DoC (Magee et al., 2014). In the first fMRI study investigating the effects of passive music listening, similar music-related activations in auditory regions were observed for healthy controls, MCS patients and one UWS patient who recovered consciousness 4 months later (Okumura et al., 2014).

Despite the overall positive effects of music interventions on behavioral responses and cognitive functions, the interpretation of these studies is difficult. Even if the brain activation of DoC patients is similar to those of healthy controls, it cannot be seen as a proof of intact awareness. It can only be concluded that a specific brain region is still capable of perceiving and processing sensory stimuli (Bruno et al., 2010). A method to detect covert awareness in patients who show no behavioral responses, are active fMRI paradigms. If patients are able to follow specific instructions, this is interpreted as a sign for a willful modulation of brain activity (Owen et al., 2006; Monti et al., 2010). However, active paradigms place high demands on the cognitive abilities of the subject, which limits their use in DoC patients. Promising alternatives are electrophysiological approaches (Naro et al., 2018) and resting-state fMRI (rs-fMRI) (Beckmann et al., 2005; Damoiseaux et al., 2006). Rs-fMRI allows the study of several different, reproducible and dynamic brain networks without external stimulation and explicit tasks (Fox and Greicius, 2010). Because they do not require active patient participation, rs-fMRI studies are particularly useful in DoC patients.

A frequently studied resting-state network (RSN) is the default mode network (DMN), which is active during rest and disabled when a task is performed. The activity of the DMN is anticorrelated with the activity of task-positive networks, e.g., the salience network, the dorsal attention network, and the executive control network (Di and Biswal, 2014). Converging evidence indicates a linear relationship between the intrinsic functional connectivity of the DMN and the level of consciousness (Vanhaudenhuyse et al., 2010; Fernández-Espejo et al., 2012; Norton et al., 2012; Huang et al., 2014), see also Hannawi et al. (2015) for a review and meta-analysis. In addition to the DMN, the auditory and executive control network show altered functional connectivity in patients with impaired consciousness (Demertzi et al., 2014; Heine et al., 2015). Heine et al. (2015) investigated the functional connectivity of RSN while DoC patients were exposed to preferred music. As a main result, enhanced functional connectivity was observed in the auditory and the dorsal attention network when music was compared to scanner noise.

The aim of the present study was to investigate the integrity of different RSN and the effects of auditory stimulation on the functional connectivity in a sample of UWS patients. Three separate rs-fMRI scans were acquired while subjects were exposed to scanner noise, preferred music, or aversive auditory stimulation. The study examined the auditory network, the default mode network, and the executive control network, which include cortical regions important for consciousness and are attributed to the processing of emotional auditory stimuli.

## Materials and Methods

### Participants

Nineteen UWS patients and 19 HC matched for age (*p* = 0.111) were enrolled. Of these, six UWS patients and one HC subject had to be excluded from the analysis due to excessive movement artifacts. Thus, data of 13 UWS patients [six women, age: Md = 55 years (IQR = 50–74)] and 18 HC [15 women, age: Md = 51 years (IQR = 47–57)] were analyzed. The level of consciousness of each patient was assessed using the German version of the Coma Recovery Scale-Revised (CRS-R) (Maurer-Karattup et al., 2010) at the day of brain imaging. Each patient was classified as UWS with CRS-R total scores ranging between 4 and 8 (Md = 7; IQR = 6–8). Table 1 shows the demographic and clinical characteristics for each patient. Brainstem (early) auditory evoked potentials indicated preserved auditory functions in all patients. Exclusion criteria were: contraindications for MRI, hearing impairment, cardiorespiratory instability, sedating medication, and lesions in target regions of analyses. In Germany, neurological patients are transferred to specialized neurological early rehabilitation centers directly after acute hospitalization. Data were acquired between February 2016 and July 2019.

**Table 1:** Demographic and clinical characteristics of DoC subjects.

**Patient**	**Gender**	**Age (years)**	**Etiology**	**Time between injury and MRI scan (days)**	**CRS-R total score**	**CRS-R subscores**
DoC-001	Male	55	Stroke	87	8	1 1 2 2 0 2
DoC-002	Male	82	TBI	31	6	1 1 2 1 0 1
DoC-003	Female	51	Stroke	36	7	1 1 2 1 0 2
DoC-004	Male	77	Anoxia	40	4	0 0 2 1 0 1
DoC-005	Female	70	Anoxia	54	8	1 1 2 2 0 2
DoC-006	Female	77	Anoxia	48	8	2 1 2 1 0 2
DoC-009	Female	48	Anoxia	33	6	1 1 2 1 0 1
DoC-010	Male	55	Tumor	46	8	2 0 2 2 0 2
DoC-011	Male	56	Anoxia	28	8	1 1 2 2 0 2
DoC-013	Female	51	Tumor	34	8	1 1 2 2 0 2
DoC-014	Male	44	TBI	19	7	1 1 2 1 0 2
DoC-015	Female	48	Anoxia	29	5	1 0 2 1 0 1
DoC-017	Male	54	Anoxia	26	7	1 0 2 2 0 2

*TBI, Traumatic Brain Injury*.

### Auditory Stimulation

A questionnaire was used to ask for preferred music of the study participants. For the patients, the questionnaire was completed by close relatives/legal representatives. Audacity recording and editing software (https://www.audacityteam.org) was used to create the auditory stimuli. Several pieces of preferred music were merged together to form an excerpt with a length of 8 min. At the beginning and at the end, there was a fading-in and a fading-out of 2 s each to avoid sudden changes. For the aversive auditory stimulation condition, two pitch-shifted versions of the preferred music stream were added to the original music stream (one version being six semi-tones below and one version being one tone above the original pitch), resulting in highly dissonant and unpleasant audios. An example of the aversive audio is provided in the Supplementary Material. The auditory stimuli of both conditions were vertically centered to 0.0 and normalized to 1.0 dB to ensure similar loudness between conditions and subjects. Prior to each rs-fMRI, subjects were instructed not to focus their thoughts on anything special, to close their eyes and to relax, without falling asleep. Instructions and auditory stimulation were presented via MR-compatible headphones (NordicNeuroLab, Bergen, Norway). All auditory stimuli were administered at 70 dB.

### MRI Data Acquisition

Structural and functional brain images were collected using a 1.5 T Magnetom Avanto MRI scanner (Siemens Medical Systems, Erlangen, Germany) with an 18-channel head coil. Three 8-min functional runs were acquired; (1) scanner noise, (2) preferred music, and (3) aversive auditory stimulation, presented in counterbalanced order. Each resting-state scan was followed by a 5-min structural scan to wash-out cross-over effects of auditory stimulation. The functional runs were acquired using a T2^*^-weighted echo-planar imaging (EPI) sequence (163 volumes, 31 axial slices, 3 × 3 × 4 mm voxel size, TR = 3,000 ms, TE = 50 ms, flip angle = 90°, matrix size = 64 × 64 mm, FOV = 192 × 192 mm). The first three volumes of each functional scan were discarded to reduce initial fluctuations of the MRI signal. 3D T1-weighted anatomical images were acquired with a magnetization-prepared rapid gradient echo (MPRAGE) method. Each anatomical image consisted of 192 sagittal slices, with 256 × 256 mm matrix size and 1 mm slice thickness.

### MRI Data Preprocessing

Preprocessing and functional connectivity analyses were performed with the CONN functional connectivity toolbox (version 18.b; www.nitrc.org/projects/conn) (Whitfield-Gabrieli and Nieto-Castanon, 2012), running in a Matlab environment. Functional images were realigned and unwarped, centered, slice-time corrected, normalized into Montreal Neurological Institute (MNI-152) space, resampled to 2 × 2 × 2 mm and smoothed with a 6 mm full-width at half-maximum Gaussian kernel. Anatomical images were segmented (gray and white matter, cerebrospinal fluid) and normalized to MNI-space. No lesion extraction or masking was performed.

Denoising of functional data included regressing out six residual head motion parameters and their first temporal derivatives, principal components of the signal from white matter and cerebrospinal fluid and movement outliers, using an anatomical component-based noise correction (aCompCor) approach (Behzadi et al., 2007). Motion outliers were identified with the Artifact Detection Toolbox (http://nitrc.org/projects/artifact_detect), using conservative settings (95th percentile in a normative sample): normalized global BOLD signal *Z*-value = 3.0 and absolute subject motion <0.5 mm. Additionally, detrending was applied to remove linear trends within each functional session. After denoising, images were band-pass filtered to 0.008–0.09 Hz to reduce low-frequency fluctuations. Further motion correction was applied using the ArtRepair toolbox for SPM (version 5b; https://www.nitrc.org/projects/art_repair/).

Participants with more than 20% of rejected volumes were excluded from further analyses (*n* = 7). Among included patients, the number of rejected volumes did not differ between conditions (*p* = 0.772) and groups (*p* = 0.406). However, the number of rejected volumes per subject was included as regressor in second-level analysis to account for repeated measures analyses.

### Functional Connectivity Analysis

Functional connectivity was analyzed using a seed-based approach, in which the mean time series of a seed region is compared with the time series of all other voxels in the brain. Based on the studies of Demertzi et al. (2015) and Heine et al. (2015), all seeds belonging to a RSN were used during analysis. Three RSNs were investigated: the auditory network, the default mode network and the executive control network. The seeds belonging to each network with the corresponding MNI coordinates are presented in Table 2. The seeds were preconfigured in the CONN software and consisted of 10 mm-diameter spheres centered at the corresponding MNI coordinates (Whitfield-Gabrieli and Nieto-Castanon, 2012). Correlation maps were generated by computing Pearson correlation coefficients between the averaged time-series of one seed with time series of each voxel in the brain. For group comparisons, the correlation coefficients were normalized using Fisher's z-transformation (Lowe et al., 1998). Main effects of condition were examined with one-sample *t*-tests for the control, preferred music and aversive auditory control condition. Group differences (HC vs. DoC) were investigated for each condition and in each RSN with two-sample *t*-tests ([1,-1]; [-1,1]). Differences between conditions (preferred music vs. aversive stimulation; preferred music vs. control; aversive stimulation vs. control) were examined within each group with paired *t*-tests ([1,-1]; [-1,1]). Possible interaction effects between group (HC vs. DoC) and condition (preferred music vs. aversive stimulation, preferred music vs. control, and aversive stimulation vs. control) were analyzed with 2 x 2 mixed ANOVAs for repeated measures ([1,-1; 1,-1], [-1,1; 1,-1], ([1,-1; -1,1], [-1,1; -1,1]). Results were considered significant when they exceeded a height threshold of *p* < 0.0009 (uncorrected) with an extent threshold of *p* < 0.05 (FWE-corrected) at cluster-level.

**Table 2:** Seeds of each resting-state network used for network and ROI-to-ROI analyses.

**Network name**	**Seeds**	**MNI coordinates**		
		**x**	**y**	**z**
Auditory network	Heschl's gyrus, left^*^	−46	−20	8
	Heschl's gyrus, right	46	−16	8
Default mode network	Posterior cingulate cortex^*^	1	−61	38
	Medial prefrontal cortex	1	55	−3
	Lateral parietal, left	−39	−77	33
	Lateral parietal, right	47	−67	29
Executive control network	Dorsolateral prefrontal cortex, left^*^	−43	33	28
	Dorsolateral prefrontal cortex, right	41	38	30
	Posterior parietal cortex, left	−46	−58	49
	Posterior parietal cortex, right	52	−52	45

* *denotes the key seed (Pearson correlation coefficients between this seed and every other seed in that network were calculated to generate a ROI-to-ROI correlation matrix)*.

To determine whether UWS patients' intrinsic functional connectivity of a specific network deviates from those of HC, the mean time series was extracted from each seed of a network and correlated with the mean time series of every other seed of that network (Table 2). Seeds and MNI coordinates used for the ROI-to-ROI analyses are provided in the CONN software (Whitfield-Gabrieli and Nieto-Castanon, 2012). Mean network connectivity (mean of all pairwise correlations within a network) was calculated and compared between groups with the Mann-Whitney *U*-test for independent samples.

## Results

### Integrity of Resting-State Networks

**Figure 1:**
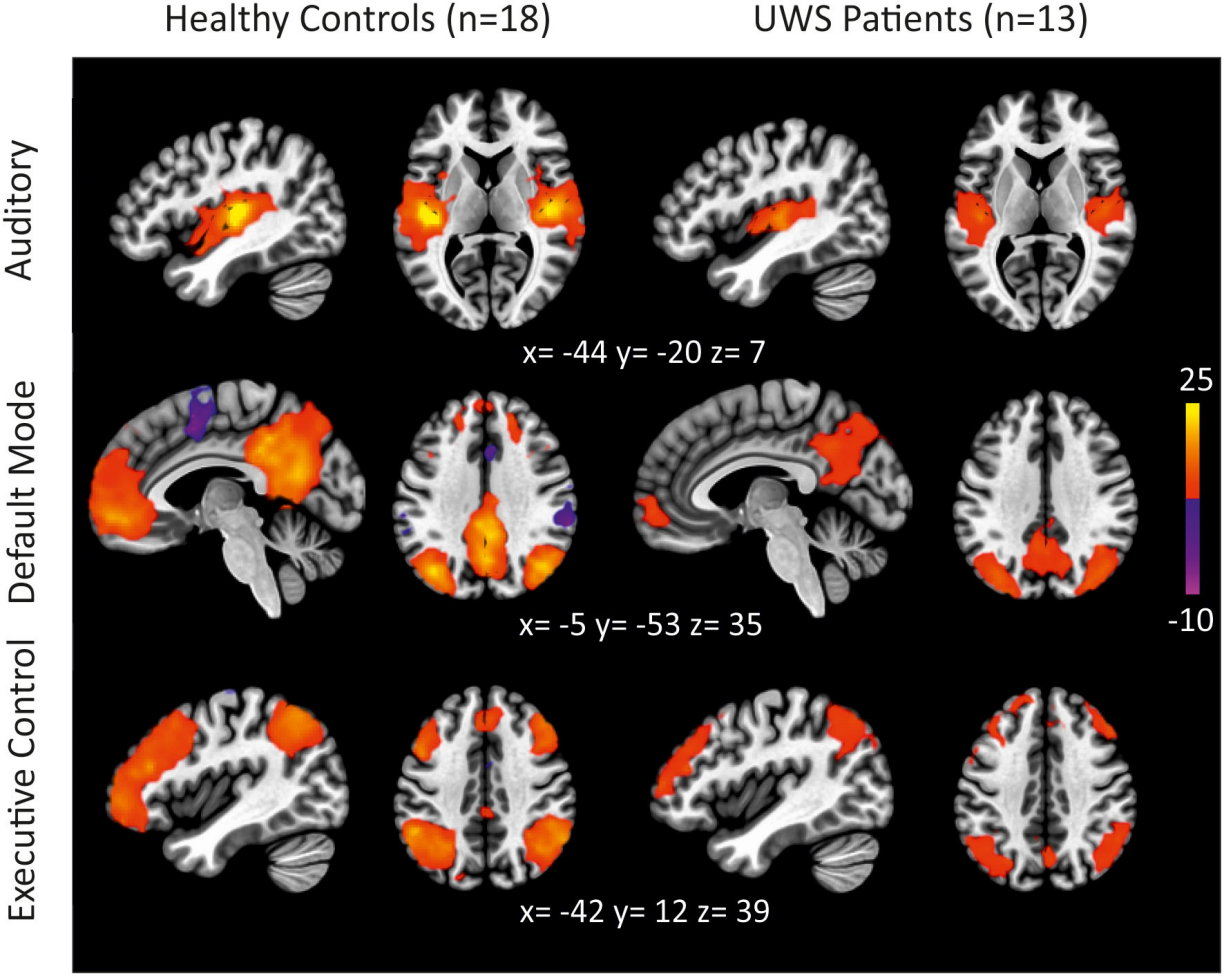
Functional connectivity in HC subjects and UWS patients during scanner noise for different RSNs (auditory, default mode, executive control) (thresholded height-level *p* < 0.0009; cluster-level FWE-corrected *p* < 0.05).

**Figure 2:**
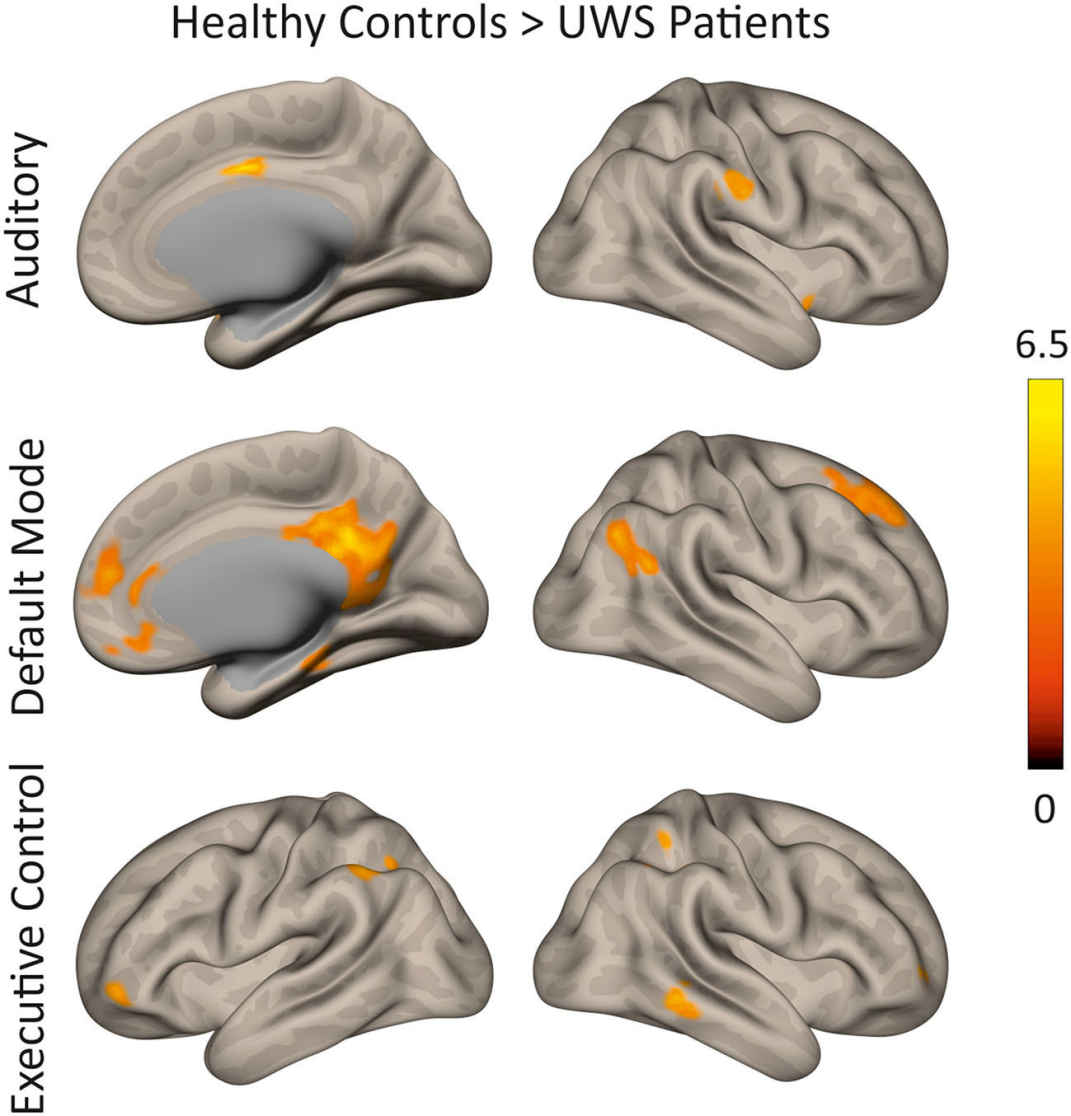
Differences in functional connectivity between HC subjects and UWS patients during scanner noise for different RSNs (auditory, default mode, executive control). Regions are shown in which HC show greater functional connectivity compared to UWS (thresholded height-level *p* < 0.0009; cluster-level FWE-corrected *p* < 0.05).

In a first step, the control condition (i.e., scanner noise) was analyzed to compare the functional connectivity of different RSN between DoC patients and HC subjects. Figure 1 presents the functional connectivity for both groups individually. Group differences are shown in Figure 2 and Table 3.

**Table 3:** Differences in functional connectivity between HC subjects and UWS patients during the control condition in different RSNs.

**Network**	**MNI Coordinates**			**Cluster size**	**Cluster p-FWE**	**p-unc peak**	**Side**	**Region**
	**x**	**y**	**Z**					
Auditory	02	−02	38	191	0.001014	0.000003	L/R	Cingulate gyrus
	54	−24	30	114	0.020235	0.000045	R	Supramarginal gyrus
	46	10	−14	97	0.042207	0.000017	L	Temporal pole
Default mode	−08	−36	30	3019	0.000000	0.000000	L/R	Posterior cingulate gyrus
	04	46	08	1068	0.000000	0.000000	L/R	Paracingulate gyrus
	28	26	50	697	0.000000	0.000001	R	Superior frontal gyrus
	−18	−18	−16	470	0.000000	0.000001	L	Parahippocampal gyrus
	42	−56	20	278	0.000056	0.000002	R	Lateral occipital cortex
	22	−32	−22	196	0.000871	0.000008	R	Parahippocampal gyrus
	−38	−74	36	174	0.001934	0.000010	L	Lateral occipital cortex
	−20	40	50	172	0.002083	0.000066	L	Superior frontal gyrus
Executive control	48	−44	54	618	0.000000	0.000007	R	Supramarginal gyrus
	−48	−46	−40	515	0.000000	0.000017	L	Lateral occipital cortex
	−04	−74	−34	260	0.000146	0.000000	L	Cerebellum
	36	58	02	149	0.006407	0.000006	R	Frontal pole
	−44	54	−04	125	0.016158	0.000021	L	Frontal pole
	60	−42	−08	123	0.017493	0.000011	R	Middle temporal gyrus

#### Auditory Network

In HC subjects, the left primary auditory cortex showed functional connectivity to areas typically associated with auditory processing. Regions ipsi- and contralaterally to the seed were activated including the Heschl's gyrus, operculum, insula, planum temporale, and polare, superior temporal gyrus, pre- and postcentral gyrus, supramarginal gyrus, supplementary motor area, putamen, cerebellum as well as cingulate and calcarine cortex. In UWS patients, similar areas were functionally connected with the left Heschl's gyrus, but the extent of this activation was reduced compared to HC. The direct comparison revealed that HC showed significantly stronger functional connectivity to anterior division of the cingulate gyrus, the right supramarginal gyrus, and the left temporal pole (cluster *p*-FWE < 0.05; *p*-unc < 0.0009).

#### Default Mode Network

The PCC was functionally connected with the precuneus, frontal pole, lateral occipital cortex/angular gyrus, and middle/superior frontal gyrus in HC. In UWS patients, functional connectivity was restricted to the PCC/precuneus and lateral occipital cortex. Compared to HC, UWS patients showed reduced connectivity to the PCC/precuneus, paracingulate gyrus/anterior cingulate cortex, the left parahippocampal gyrus, the lateral occipital cortex, and the superior frontal gyrus (cluster *p*-FWE < 0.05; *p*-unc < 0.0009).

#### Executive Control Network

The executive control network encompassed bilaterally the dorsolateral prefrontal cortex and frontal eye fields as well as the bilateral intraparietal sulcus and superior parietal lobule, the middle temporal gyrus, the frontal pole and the superior frontal/paracingulate gyrus in HC. In UWS patients, the left dorsolateral prefrontal cortex was connected to each of the four core regions as well, but the extent was reduced. More specifically, connectivity was reduced to the left lateral occipital cortex, the left frontal pole and left cerebellar regions (cluster *p*-FWE < 0.05; *p*-unc < 0.0009). In the right hemisphere, connectivity was reduced to the supramarginal gyrus, the middle temporal gyrus and the frontal pole (cluster *p*-FWE < 0.05; *p*-unc < 0.0009).

### ROI-to-ROI-Analyses

**Figure 3:**
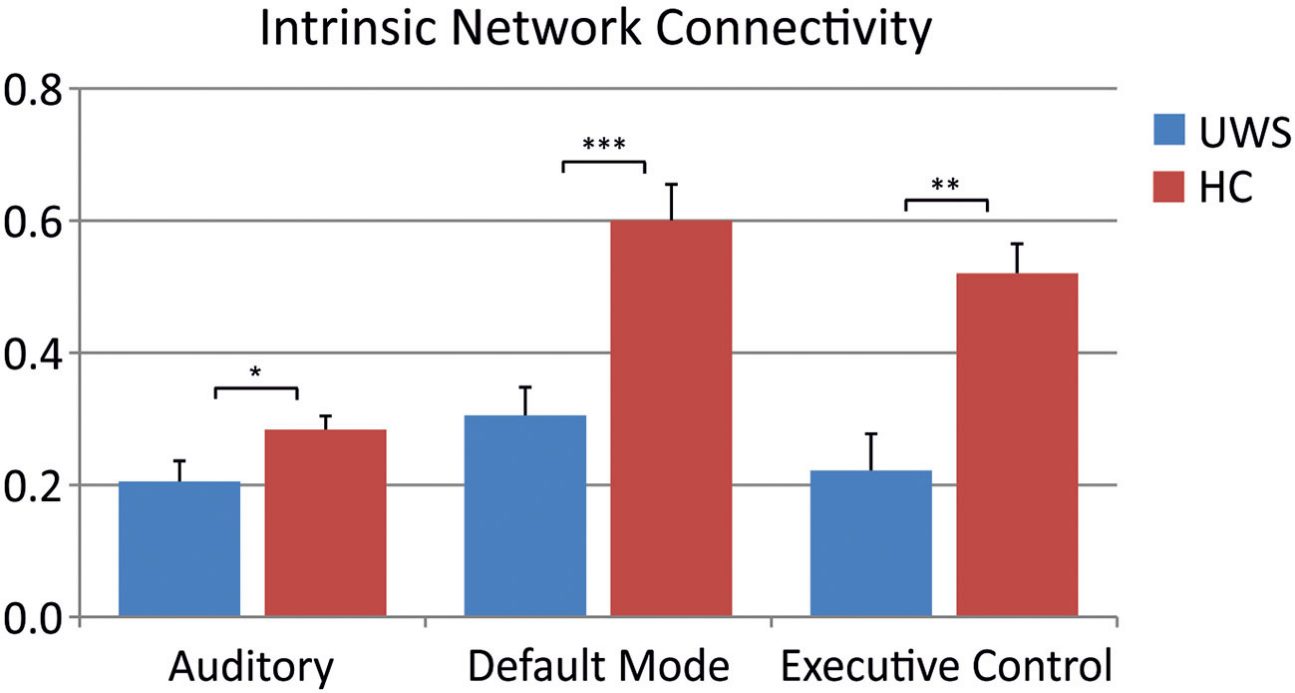
Comparison of intrinsic network connectivity between HC subjects and UWS patients during scanner noise for different RSNs (Mann-Whitney *U*-test: *p < 0.05; **p < 0.01; ***p < 0.001).

The intrinsic connectivity of the auditory network [*F*_(1, 29)_ = 4.88; *p* < 0.05], the DMN [*F*_(1, 29)_ = 16.07; *p* < 0.001], and the executive control network [*F*_(1, 29)_ = 12.236; *p* < 0.01] differed between UWS patients and HC, see Figure 3. There was no relationship between the level of consciousness, as measured with the CRS-R, and the intrinsic connectivity of the different resting-state networks.

### Modulation of Functional Connectivity Through Auditory Stimulation

**Figure 4:**
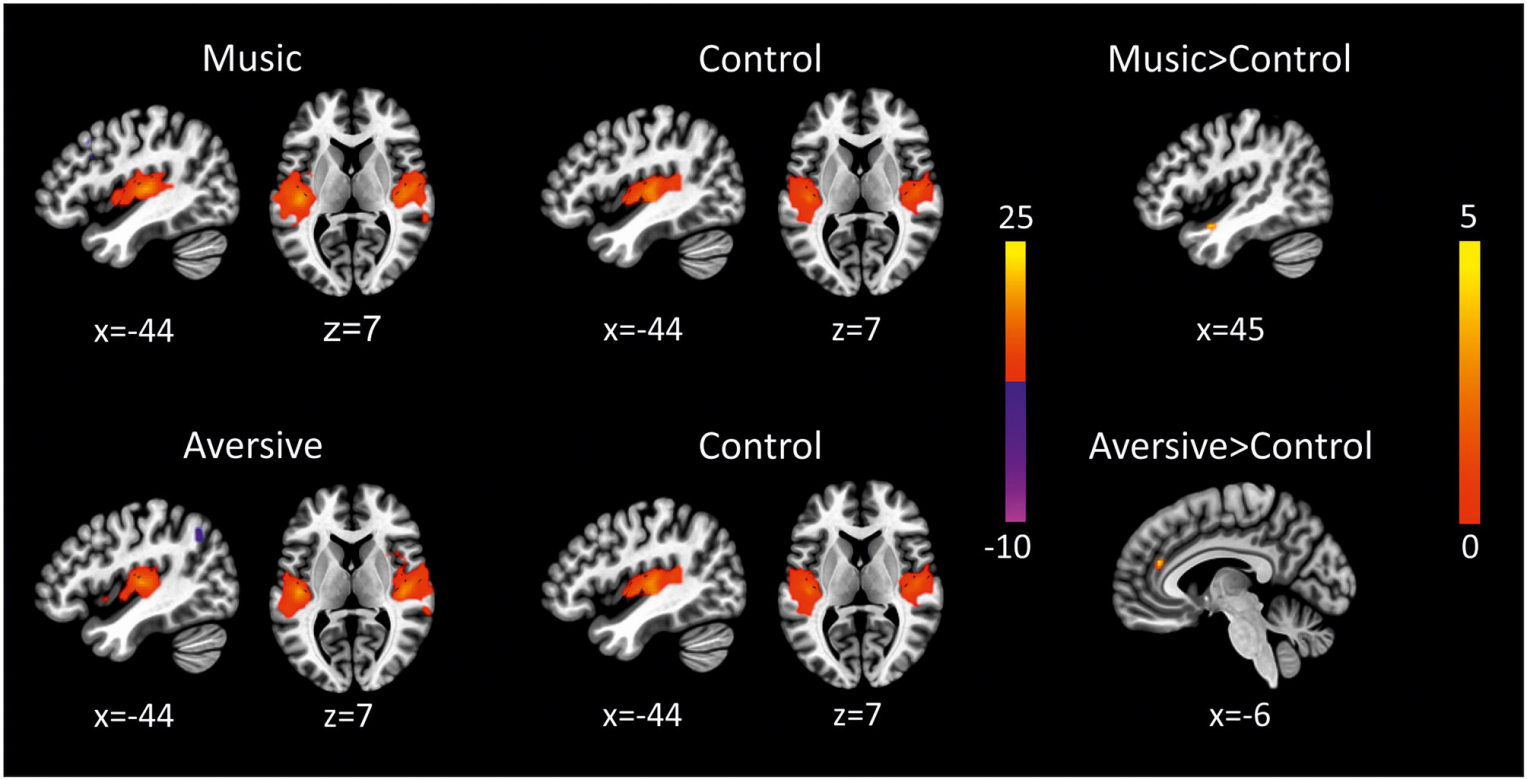
Alterations of resting-state functional connectivity in UWS patients in the auditory network (preferred music > control condition and aversive auditory stimulation > control condition), (thresholded height-level *p* < 0.0009; cluster-level FWE-corrected *p* < 0.05).

In HC, there were no differences between conditions across all RSN. In UWS patients, alterations in functional connectivity were observed in the auditory network (Figure 4 and Table 4). Functional connectivity was increased to the right insular cortex and the cingulate gyrus in the auditory network when aversive auditory stimulation was compared to scanner noise (cluster *p*-FWE < 0.05; *p*-unc < 0.0009). When preferred music was compared to scanner noise, functional connectivity to the right planum polare was increased (cluster *p*-FWE < 0.05; *p*-unc < 0.0009).

**Table 4:** Alterations of resting-state functional connectivity in UWS patients in the auditory network.

**Contrast**	**MNI coordinates**			**Cluster size**	**Cluster p-FWE**	**p-unc peak**	**Side**	**Region**
	**x**	**y**	**z**					
Aversive> Control	36	02	−20	91	0.002668	0.000004	R	Insular cortex
	−04	40	18	75	0.004428	0.000003	L/R	Cingulate gyrus
Music> Control	44	02	−18	70	0.011396	0.000017	R	Planum polare

## Discussion

This study investigated the integrity of different RSNs in UWS patients and whether the functional connectivity may be modulated through auditory stimulation.

### Integrity of Resting-State Networks

Being exposed to scanner noise, UWS patients showed reduced functional connectivity in all investigated RSNs compared to healthy control subjects. This finding is in line with several studies, proving that the intrinsic functional connectivity of different RSN is disrupted in patients with DoC. The connectivity of the DMN, for example, is significantly reduced (Vanhaudenhuyse et al., 2010; Crone et al., 2015; Di Perri et al., 2016) and discriminates UWS from MCS (Demertzi et al., 2015). In addition, the decreased DMN connectivity is reported to correlate with CRS-R scores (Rosazza et al., 2016).

The intrinsic connectivity of the DMN has further been shown to predict whether patients recover consciousness (Qin et al., 2015; Wu et al., 2015). In particular, the DMN inter-network anticorrelation (i.e., negative correlations between the DMN and task-positive networks) has been proven to be an important predictor for outcome (Song et al., 2018). However, while most studies investigate patients with chronic DoC, Threlkeld et al. (2018) have shown that both, DMN correlations and anticorrelations, are disrupted in patients with acute traumatic brain injuries and normalize when patients recover consciousness (Threlkeld et al., 2018). Therefore, follow-up examinations of clinical assessments are necessary to investigate the prognostic value of the DMN connectivity for the recovery of patients with DoC. In addition, future studies should focus on the question whether and to what extent the observed alterations change over time.

Despite the large number of studies emphasizing the relationship between the DMN and consciousness, alterations of other RSN have also been reported. The present study revealed significant differences between UWS patients and HC subjects in the auditory network and the executive control network. In a study of Demertzi and colleagues, the integrity of the auditory network had the highest accuracy in discriminating UWS and MCS patients, allowing automatic classification (Demertzi et al., 2015). According to previous studies, the functional connectivity strength of the executive control network is reduced in DoC, although no significant difference between UWS and MCS was found (Qin et al., 2015; Wu et al., 2015). Therefore, the exact role of the executive control network in constituting the level of consciousness needs to be further investigated in future studies (Qin et al., 2015).

### Modulation of Functional Connectivity Through Auditory Stimulation

Being exposed to musical stimulation during rs-fMRI had no effect on cerebral responses in HC. This finding is in line with previous studies (Castro et al., 2015; Heine et al., 2015) and might be due to the fact that cerebral responses in HC are already at high level (Heine et al., 2015).

Preferred music increased functional connectivity in the auditory network: there was higher connectivity to the right planum temporale when preferred music was compared to scanner noise. The identified cluster is part of the primary auditory cortex, indicating that this region is stronger activated when patients are exposed to preferred music compared to scanner noise. Previous studies have also shown alterations of functional connectivity in response to preferred music. Heine et al. (2015) for example demonstrated increased functional connectivity to the precentral gyrus and the left dorsolateral prefrontal cortex when preferred music was compared to scanner noise. Using EEG, Castro and colleagues demonstrated that listening to preferred music enhances event-related responses to a highly significant stimulus (i.e., patient's own name) compared to a control condition (Castro et al., 2015).

Aversive auditory stimulation was associated with subtle changes of functional connectivity in the auditory network, too. There was higher functional connectivity to the insular cortex and the anterior cingulate gyrus during aversive auditory stimulation compared to scanner noise. Hence, the insular cortex, as part of the primary auditory cortex, was stronger activated when patients listened to aversive auditory stimulation compared to the control condition, in which patients were exposed to scanner noise. In addition, activity of the anterior part of the cingulate gyrus was increased during aversive auditory stimulation. When patients are exposed to auditory stimulation, arousal and attention are increased and the thalamus, anterior cingulate gyrus and the dorsolateral prefrontal cortex are activated (Rollnik and Altenmüller, 2014). Thus, the observed increased activity of the anterior cingulate gyrus suggests that attention and/or arousal of UWS patients was promoted through the aversive auditory stimulation.

Although more data are needed to prove this view, the findings of the present study suggest that stimulation with either pleasant or unpleasant auditory stimuli might modulate brain activations in UWS patients. This might be due to the fact that UWS patients need particular strong stimuli to be activated. The most salient brain activations in subjects with severe brain injuries are evoked by emotional stimuli such as the subject's own name (Wu et al., 2018), familiar voices (Di et al., 2007), preferred music (Okumura et al., 2014; Heine et al., 2015), and emotional sounds (Gao et al., 2019). The aversive auditory stimulation implemented in the present study represents a highly salient stimulus which is suitable to elicit responses in brain regions that have been linked to the processing of auditory stimuli and attention/arousal. However, the observed modulation of brain activation does not explicitly show how the patients perceive and experience the emotional stimuli (Yu et al., 2013).

The positive effects of stimuli with high emotional valence are attributed to an environmental enrichment of the patient which increases the arousal level and attention of the patient (Rollnik and Altenmüller, 2014). This effect is used in sensory stimulation programs, which are supposed to activate neural networks, boost brain plasticity, and avoid sensory deprivation (Wu et al., 2018). In the present study, preferred music and aversive auditory stimulation modulated brain activity, suggesting that these types of auditory stimulation have the potential to trigger cerebral responses in patients with impaired consciousness. Preferred music should therefore be used in the context of sensory stimulation programs to enhance responsiveness during rehabilitative treatment. Hence, it is strongly recommended that sensory stimulation programs applied during rehabilitative treatment include personally relevant and emotional stimuli, with different degrees of emotional valence according to the individual level of consciousness.

Although our results are in line with previous studies, demonstrating modulation of brain activity through auditory stimulation, the reported brain structures are only partially identical. These discrepancies might be explained by different control conditions used across studies. While Heine et al. (2015), for example, contrasted preferred music to scanner noise (Heine et al., 2015), the present study used scanner noise as well as aversive auditory stimulation as control conditions for patients' preferred music. For the aversive auditory stimulation condition, the preferred music stream was electronically manipulated in a way that it was supposed to be perceived as dissonant while basic acoustical features were maintained. The results of the present study suggest that the applied modification of the preferred music might have the potential to modulate functional connectivity because of its high emotional valence. Another difference relates to the composition of the study sample. In studies showing positive effects of preferred music, either individual cases (Okumura et al., 2014) or samples comprising UWS and MCS patients (Heine et al., 2015) were included. Since our clinical sample consisted of UWS patients only, it is likely that stimuli needed to have a higher emotional valence to elicit similar cerebral responses reported in other studies. This finding is also reflected in the observation that close family members of the patients tend to report a higher level of responsiveness than clinicians (Moretta et al., 2017). On the one hand this could be due to an optimistic bias, but it could also be attributed to different types of interaction both groups employ. While neutral stimuli are usually used during clinical assessments, family members typically address the patient on a more personal level, for example by using their name or their favorite objects and referring to shared memories (Magliacano et al., 2019).

## Conclusion

Resting-state functional connectivity is severely impaired in UWS patients. This was demonstrated for each network associated with consciousness (i.e., auditory, default mode, and executive control network). Preferred music and aversive auditory stimulation modulated activity of the auditory network in UWS patients. These results add to the findings that UWS patients need strong stimuli to elicit cerebral responses. This should be considered in sensory stimulation programs, which are applied to improve cognitive and behavioral responses and to recover consciousness.

## Limitations

There are some limitations that need to be addressed. First, the level of consciousness was assessed only once at the day of brain imaging, although a study by Wannez et al. (2017) shows that at least five separate CRS-R assessments over a period of two weeks are necessary to establish an accurate diagnosis. In view of the large fluctuations in patients with DoC, it is possible that no signs of awareness were detected in some patients, even though they might have been present. It can therefore not be excluded that some diagnoses of the level of consciousness are incorrect. In addition, preprocessing and functional connectivity analyses of the rs-fMRI data might have been influenced by the brain lesions of the patients, which considerably varied in size and location. One possibility might have been to mask focal brain lesion to ensure that the signal used for statistical inferences originates from neuronal components only. The normalization of functional and structural MRI images to an MNI template image is an important step during preprocessing and the output of this step is crucial for the quality of the data. In the present study, a non-linear spatial normalization approach was used, although lesioned images might require more sophisticated measures (Sitaram et al., 2019). Another limitation relates to the variety of diagnoses of patients included in the study. However, different injuries can cause DoC and most previous studies included heterogeneous study samples. Due to the different prognoses of traumatic and non-traumatic brain injuries, at least these two entities should be examined separately or compared to each other in future studies.

## Data Availability Statement

The raw data supporting the conclusions of this article will be made available by the authors, without undue reservation.

## Ethics Statement

The studies involving human participants were reviewed and approved by Ethics Committee of Hannover Medical School. The patients/participants provided their written informed consent to participate in this study.

## Author Contributions

MB designed and conceptualized the study, acquired the data, performed the data analysis, and drafted the manuscript. SS, CG, JK, MS, GH, C-WW, TM, and JR interpreted the data and critically revised the manuscript for intellectual content. All authors read and approved the final manuscript.

## Conflict of Interest

The authors declare that the research was conducted in the absence of any commercial or financial relationships that could be construed as a potential conflict of interest.

## Abbreviations

CRS-R: Coma Recovery Scale-Revised
DOC: Disorders of consciousness
DMN: Default mode network
fMRI: Functional magnetic resonance imaging
HC: Healthy controls
MCS: Minimally conscious state
PCC: Posterior cingulate cortex
RSN: Resting-state network
UWS: Unresponsive wakefulness syndrome.
